# Cryo-EM structures and functional characterization of homo- and heteropolymers of human ferritin variants

**DOI:** 10.1038/s41598-020-77717-4

**Published:** 2020-11-26

**Authors:** Jose Irimia-Dominguez, Chen Sun, Kunpeng Li, Barry B. Muhoberac, Grace I. Hallinan, Holly J. Garringer, Bernardino Ghetti, Wen Jiang, Ruben Vidal

**Affiliations:** 1 Department of Pathology and Laboratory Medicine, Indiana University School of Medicine, 635 Barnhill Dr., MSB A136, Indianapolis, IN 46202 USA; 2 Department of Biological Sciences, Markey Center for Structural Biology, Purdue University, West Lafayette, IN 47906 USA; 3 Department of Chemistry and Chemical Biology, Indiana University-Purdue University Indianapolis, Indianapolis, IN 46202 USA; 4 Stark Neurosciences Research Institute, Indiana University School of Medicine, Indianapolis, IN 46202 USA; 5 Present Address: Department of Molecular and Cellular Endocrinology, Beckman Research Institute, City of Hope, 1500 E. Duarte Road, Gonda North, Bay 3131E, Duarte, CA 91010 USA

**Keywords:** Neurology, Neurological disorders, Alzheimer's disease

## Abstract

The role of abnormal brain iron metabolism in neurodegenerative diseases is still insufficiently understood. Here, we investigate the molecular basis of the neurodegenerative disease hereditary ferritinopathy (HF), in which dysregulation of brain iron homeostasis is the primary cause of neurodegeneration. We mutagenized ferritin’s three-fold pores (3FPs), i.e. the main entry route for iron, to investigate ferritin’s iron management when iron must traverse the protein shell through the disrupted four-fold pores (4FPs) generated by mutations in the ferritin light chain (FtL) gene in HF. We assessed the structure and properties of ferritins using cryo-electron microscopy and a range of functional analyses in vitro. Loss of 3FP function did not alter ferritin structure but led to a decrease in protein solubility and iron storage. Abnormal 4FPs acted as alternate routes for iron entry and exit in the absence of functional 3FPs, further reducing ferritin iron-storage capacity. Importantly, even a small number of MtFtL subunits significantly compromises ferritin solubility and function, providing a rationale for the presence of ferritin aggregates in cell types expressing different levels of FtLs in patients with HF. These findings led us to discuss whether modifying pores could be used as a pharmacological target in HF.

## Introduction

Abnormal iron metabolism is observed in many neurodegenerative diseases. The dysregulation of brain iron homeostasis, occurring as the primary cause of neurodegeneration, is currently only observed in hereditary ferritinopathy (HF), aceruloplasminemia, and the recently reported autosomal recessive *IREB2*-related disorder^1–4^. HF, also known as neuroferritinopathy, is an autosomal dominant, adult onset, degenerative disease caused by mutations in the *ferritin light chain* (*FtL*) gene^3,5^. The clinical phenotype of HF is characterized by a progressive movement disorder, behavioral disturbances, and cognitive impairment^3^. The main neuropathologic findings are cystic cavitation of the basal ganglia, substantial iron deposition, and ferritin inclusion bodies (IBs) composed by wild-type FtL (wtFtL), mutant FtL (MtFtL), and ferritin heavy chain (FtH) subunits^6^. Ferritin IBs are not limited to the central nervous system (CNS); in fact, IBs have also been demonstrated in cells of several organs, including skin, kidney, liver, and muscle^3,6^. In vitro and in vivo studies have shown that MtFtL polypeptides form soluble ferritin homopolymers, and heteropolymers with wtFtL and FtH subunits. The presence of MtFtL subunits in ferritin polymers leads to a specific structural disruption that may explain the reduced ability of ferritin to incorporate and retain iron, enhanced oxidative damage, and the aggregation of ferritin during iron loading^7–13^.

Ferritin is the main cellular iron storage protein and plays a pivotal role in iron homeostasis. Ferritin is a 24-subunit heteropolymer with different ratios of FtL and FtH subunits. The FtH subunit has ferroxidase activity while the FtL subunit has a predominant role in iron storage^3^. Homopolymers containing only FtL occur in vivo^14^, but incorporate iron more slowly than heteropolymers^14,15^. FtL and FtH polypeptides are 54% identical in amino acid sequence with specific amino acid differences related to function, but with similar tertiary structures making them interchangeable within the ferritin molecule. Mammalian ferritin forms an ~ 480 kDa spherical shell (~ 12 nm of outer and 7 nm inner diameter) from 24 self-assembling subunits creating eight three-fold pores (3FP) and six smaller four-fold pores (4FP) (Supplementary Fig. S1a,b)^3,14,16^. Ferritin oxidizes ferrous iron (Fe^2+^) to ferric iron (Fe^3+^), which is then sequestered within its interior as an iron-oxy biomineral matrix of up to 4500 iron atoms. Each subunit is composed of a bundle of 4 parallel α helices (A, B, C, D), a C terminus with a shorter α helix (E) and a long loop (L) connecting helices B and C (Supplementary Fig. S1c)^16,17^. Extensive helix–helix interactions occur between these bundles and between the inwardly pointing E-helices donated from 4 subunits forming the 4FPs^16,17^. The subunits are structurally related by 4-, 3-, and 2-fold symmetry axes and pack tightly together except at the 3- and 4-fold axes where they form the narrow pores traversing the shell. Detailed structural information about ferritins is available since ferritin crystals are obtained relatively easily^9,17^. Residues around the eight 3FP pores are conserved, with predominantly hydrophilic amino acids, and have been proposed as the main entry route for iron and the oxidation sites for iron^18^. Two residues, Asp-128 and Glu-131 in 3FPs, are highly conserved in all mammalian ferritins. Changes in these amino acids that modify the hydrophilicity of the area result in a significant slowdown in the rates of iron uptake^17–20^. In contrast, 4FPs are smaller, lined with hydrophobic residues and closed to iron transit. It has been suggested that 4FPs form a “proton wire”, providing an exit pathway for protons during iron mineralization^21^. Mutations of the amino acid residues lining the 4FPs are known to be able to destabilize ferritin polymers and interfere with ferritin iron deposition^22^.

The structure and biology of ferritin’s 4FPs became of particular interest after nucleotide duplications in exon 4 of the *FtL* gene were found in individuals with HF^3^. These mutations in *FtL* alter the C-terminal sequence and length of the polypeptide, disrupting 4FPs and causing loss-of-ferritin function by iron mishandling through attenuated iron incorporation in ferritin, without affecting 3FPs^7,9^. The primary structural difference between wtFtL and the MtFtL polypeptides associated with HF is the loss of the E-helices around 4FPs (Supplementary Fig. S1d) in the MtFtL^9^. It has been proposed that the unraveling and extension of the C-terminal portion of the MtFtL subunits allow ferritin shells to link with unraveled C-termini on other shells through iron ion bridging leading to a gain-of-toxic function by aggregation of ferritin and IB formation^9^. The disruption of the 4FPs and loss-of-normal ferritin function triggers intracellular iron accumulation and over-production of ferritin polypeptides, creating a positive feedback loop that leads to the formation of ferritin IBs^3^.

Herein, we mutated key residues in the 3FP to block the flux of iron through this route. Previous work studied the impact of disrupted 4FP on ferritin stability and function^7–13^, but whether the abnormal 4FP could act as an alternative route for iron uptake and/or exit remains unknown. We used cryo-electron microscopy (cryo-EM) to solve the structure of ferritins with disrupted 4FPs and with modified main entry/exit routes for iron around the 3FP. We also performed functional studies in ferritin homo- and heteropolymers to better understand the transfer of Fe^2+^ ions in and out of the protein shell with intact or modified 3FPs and 4FPs and determined the minimal amount of mutant isoform that leads to a significant decrease in ferritin function. Modifying pores could be used as a potential target for pharmacological use in HF as it has been initially assessed in mini-ferritin (Dps protein), for which selective small-molecule inhibitors have been developed that interact with active sites around the 3FP and modify iron availability^17,23,24^.

## Results and discussion

### Cryo-EM shows significant disruption of ferritin 4FPs and function in mutant FtLs

**Figure 1:**
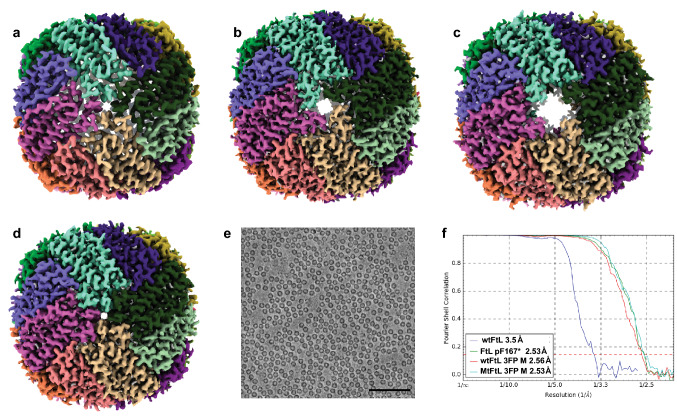
Structure of ferritins. Density maps of the ferritin homopolymeric apoferritins (24-subunit) colored by chain. Wild-type FtL (**a**), FtL p.F167* (**b**), MtFtL with the 3FP double mutation (**c**), and wtFtL with the 3FP double mutation (**d**) viewed down one of the fourfold axes. Image representation of the ferritin MtFtL with the 3FP double mutation mutant dataset (**e**). Gold Standard Fourier shell correlation (FSC) curves of the four datasets (**f**) showing the global resolution of 3.5 Å for wtFtL (blue), 2.53 Å for FtL p.F167* (green), 2.56 Å for wtFtL 3FPM (red), and 2.53 Å for MtFtL 3FPM (light blue). Scale bar 100 nm. The figure was generated using Adobe Illustrator CC2019 23.0.3 https://www.adobe.com/products/illustrator.html.

**Figure 2:**
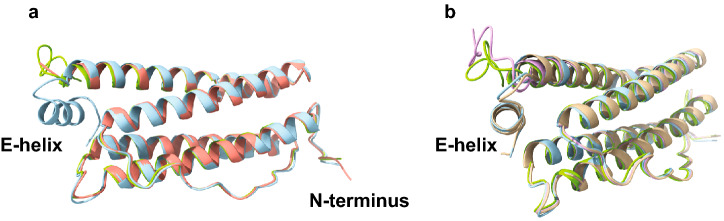
Ribbon diagrams. (**a**) Superposition of the structures of recombinant wtFtL (light blue) and FtL p.F167* (green) identified by cryo-EM. The MtFtL (red) structure is derived from PDB 4V6B. Subunits are folded as 4-helix bundles with a fifth short helix inclined at ~ 60° to the four-helix bundle. The E-helix is missing in the mutant (red), remaining unaccounted for crystallographically^9^. Conformational differences between wtFtL and MtFtL were seen to reach back to the D-helix (residue Leu-155)^9^. (**b**) Superposition of the structures of recombinant wtFtL (light blue), wtFtL with 3FP mutations (brown), FtL p.F167* (green), and MtFtL with 3FP mutations (light pink) obtained by cryo-EM. We are able to observe the C-terminus of the MtFtL subunit that was not accounted for crystallographically. Interestingly, the C-terminus of MtFtL and FtL p.F167* adopt opposite orientation compared to wtFtL. No differences were observed between the wtFtL (light blue) and the wtFtL with 3FP mutations (brown). The figure was generated using Adobe Illustrator CC2019 23.0.3 https://www.adobe.com/products/illustrator.html.

Mutations affecting the E-helix of FtL lead to iron mishandling and to an abnormal accumulation of ferritin in HF^3^. The mutant FtL p.Phe167SerfsX26 (MtFtL) (Supplementary Fig. S1d) has been previously shown to assemble into soluble 24-mer homopolymeric shells that are very similar in size, spherical shape, and overall structure to wtFtL homopolymers^7,9^. Since the crystallographic structure of MtFtL homopolymers showed a remarkable disruption of the MtFtL C-terminal helices^9^, we investigated the role of the C-terminus of FtL in the structure and function of ferritin. To this aim, we generated recombinant apoferritin homopolymers with a premature stop codon (FtL p.F167*), in which all mutated residues in MtFtL were removed Supplementary Fig. S1d)^9^. The FtL p.F167* polypeptides self-assembled into soluble homopolymers as determined by native acrylamide gel electrophoresis (Supplementary Fig. S1e) and transmission electron microscopy (TEM) studies (not shown). Cryo-EM analysis of homopolymers composed by FtL p.F167* apoferritins shows significant disruption of the 4FPs (Fig. 1), while the WtFtL and FtL p.F167* subunits adopt essentially the same fold (Fig. 2). The loop between helices D and E, which may be disordered in wtFtL^9^, is clearly visible by cryo-EM. The major differences reside in the missing DE turn (amino acids 152–161) and lack of the E helix in the FtL p.F167* subunit (Fig. 2), as reported for MtFtL by X-ray crystallography^9^.

**Figure 3:**
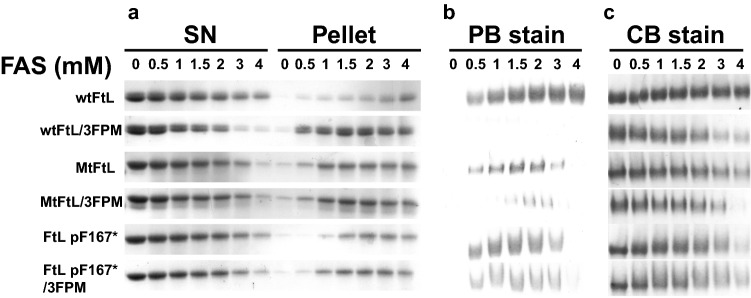
Solubility of ferritin homopolymers upon iron loading and Iron uptake. (**a**) Apoferritin homopolymers (1 μM) were iron loaded by incubating the protein with increasing amounts of ferrous ammonium sulfate (FAS) (0–4 mM) in HEPES buffer (pH 7.4) for 2 h. The soluble and insoluble fractions were separated by centrifugation, resolved by SDS-PAGE and stained with coomassie blue (CB). To assess iron uptake, ferritin homopolymers were iron-loaded for 2 h at pH 7.40. The soluble iron loaded homopolymers were resolved in a native gel and the iron content monitored by Prussian blue (PB) staining (**b**). Total protein was assessed by staining with CB (**c**). SN indicates supernatants (soluble fraction). The figure was generated using Adobe Photoshop CC2019 20.0.4 and Adobe Illustrator CC2019 23.0.3 https://www.adobe.com/products/illustrator.html.

**Figure 4:**
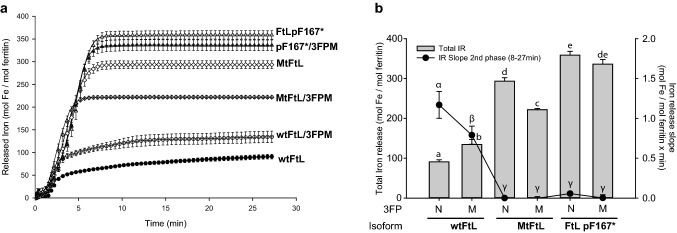
Iron release from ferritin homopolymers. (**a**) Ferritin homopolymers were iron loaded (1:500 ratio) and the release of iron (IR) with the reductants NADH/FMN was monitored spectrophotometrically following the formation of the bipyridyl:Iron complex for 27 min at 520 nm, then plotted from 5–9 independent experiments per isoform. (**b**)Total iron release is represented as a histogram, left axis. After an initial rapid release of iron, the slope of the slow iron release phase was calculated from minute 8 to 27 and represented as a line plot, right axis. Results are presented as mean ± SEM. Groups with no coincident letters (**a–e** for total IR and α-γ for IR slope of the 2nd phase) are statistically different (p < 0.05). The figure was generated using Statgraphics Centurion XV v.15.1.02 https://www.statgraphics.com/ and SigmaPlot for Windows v.12.5 Build 12.5.0.38 http://www.sigmaplot.com.

To assess iron loading function, FtL p.F167* apoferritins were loaded from 0 to 4000 atoms of Fe^2+^ as ferrous ammonium sulfate (FAS) per molecule of ferritin (24-mer). The soluble iron-loaded protein fraction was separated from the insoluble protein and the soluble portion was calculated. The increase in iron concentration led to a decrease in the solubility of FtL p.F167* ferritins, similar to what was observed in MtFtL homopolymers (Fig. 3a, Supplementary Fig. S2a). As previously reported^7,9^, the uptake of iron by wtFtL ferritins plateaued at ~ 4000 Fe^2+^:ferritin, while homopolymers of the MtFtL associated with HF (p.Phe167SerfsX26) showed a severely impaired iron uptake, peaking at ~ 1500 Fe^2+^:ferritin (Fig. 3b,c, Supplementary Fig. S2f,h). Interestingly, FtLp.F167* ferritins showed an iron mineralization capacity between the wtFtL and the MtFtL ferritins, peaking at ~ 2000:1 Fe^2+^:ferritin (Fig. 3b,c, Supplementary Fig. S2). We also evaluated the iron release kinetics by loading 500 atoms of Fe^2+^ per ferritin homopolymer molecule. The soluble protein was treated with reducing agents and bipyridil to force the release of iron from the mineralized core. Compared to the wtFtL, iron released was highly increased in the MtFtL and FtL p.F167* homopolymers (Fig. 4), suggesting that a normal 4FP structure is needed to prevent iron leaking from the mineralized core of the ferritin protein, while an open pore allows the access of reducing agents and the fast removal of iron from inside the protein nanocage^24^. Small changes in pH seem to modify significantly iron storage when the 4FP is compromised. Compared to the wtFtL, we observed a dramatic decrease in iron storage capacity in the MtFtL and FtL p.F167* homopolymers, even by small changes in the pH, such as from 7.4 to 7.2 without major changes in protein solubility (Supplementary Fig. S3). These changes in function may be due to a decreased capacity of the mutant nanocage to mineralize and retain iron at the protein core due to the disruption of the 4FP. Other studies have shown that changes in pH compromise FtH homopolymer’s function retaining iron in the mineralized core^25^, highlighting the importance of pH in the functionality of ferritin relatively to the uptake of iron and its mineralization. The main exit of iron from the ferritin mineral core is believed to involve ferritin degradation in the lysosome^26^, where ferritin is transferred in a highly regulated process known as ferritinophagy^27^. Ferritin containing MtFtL may be more sensitive to small pH changes in autophagy intermediates^28^, increasing the labile iron pool (LIP) within the autophagy pathway. Increases in LIP may lead to oxidative damage of organelles from the early autophagosomes, impairment of cellular autophagy, and cell dysfunction.

### Mutagenesis of the three-fold pores causes narrowing of the pores and severe impairing of ferritin function in the presence of abnormal four-fold pores

**Figure 5:**
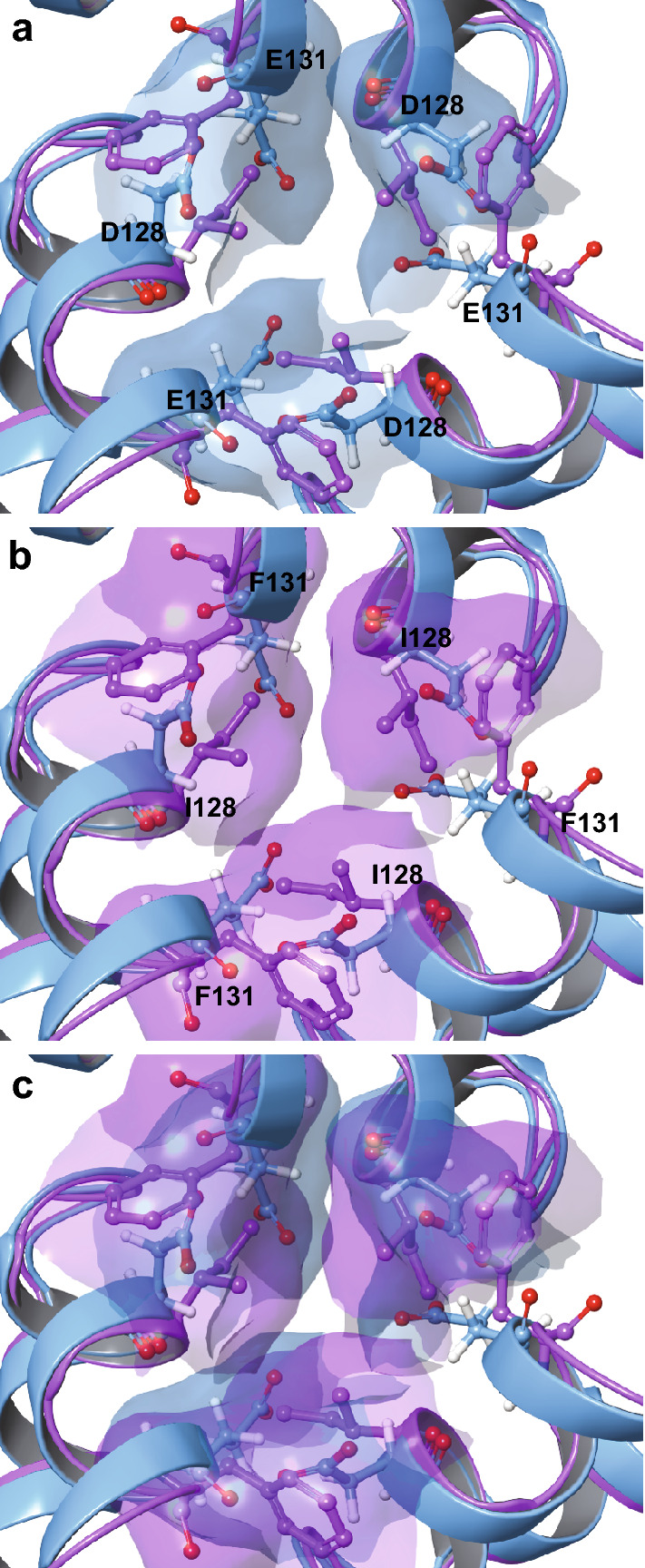
Superposition of recombinant wtFtL and wtFtL/3FPM at the three-fold-axis. The wt- (faded violet) and wtFtL/3FPM (light blue) structures are represented using a ribbon diagram. Residues D128 and E131 are highlighted in the wtFtL, while residues I128 and F131 are highlighted in the wtFtL/3FPM apoferritins. The surface of the wtFtL with the D128 and E131 amino acids is colored in blue (**a**) while the surface of the wtFtL/3FPM with the mutant amino acids is colored in violet (**b**). A superposition of (**a**) and (**b**) is shown in (**c**). The figure was generated using Adobe Illustrator CC2019 23.0.3 https://www.adobe.com/products/illustrator.html.

Negatively charged residues (D128 and E131) (Supplementary Fig. S1d) facing the 3FP channels generate a negative electrostatic gradient capable of guiding iron ions towards the ferroxidase centers of 24-subunit ferritins. Three-fold pores are used for iron entry to and exit from the protein cage in vitro^24,29^. X-ray crystallography of ferritin co-crystallized with Co^2+^, modeling Fe^2+^, show multiple metal ions aligned within the ion channels, similar to membrane ion channel proteins^30^. Numerous studies have assessed the effect(s) of mutagenesis of different residues along the 3FP channel, focusing on iron entry, mineral formation, and iron release from ferritin^31^. We focused on residues D128 and E131 of FtL to assess the roles of 3FPs in iron uptake/released when the 4FPs are compromised. Each of the 3FP-forming subunits donated one Asp-128 and one Glu-131 resulting in a six-coordinate metal site close to the interior surface of the protein. We mutagenized residues 128 (D128I) and 131 (E131F) producing recombinant wtFtL, FtL p.F167*, and MtFtl ferritin with the D187I and E131F changes (3FPM). All three polypeptides self-assembled into soluble homopolymers as determined by native acrylamide gel electrophoresis and TEM (Supplementary Fig. S1). Interestingly, as compared to wtFtL, the p.F167* homopolymers with the D128I and E131F mutations (FtL p.F167*/3FPM) had a diameter that was on average ~ 0.7 nm smaller (not shown). Cryo-EM structural analysis of apo-homopolymers composed of wtFtL and wtFtL with the D128I and E131F mutations (wtFtL/3FPM) showed significant differences in the density of the residues lining the narrowest part of the 3FPs (caused by the substitutions), but did not show any loss of α-helix or any physical consequence of pore modification (Fig. 5).

During iron loading experiments, we observed that narrowing of the 3FPs decreased ferritin solubility, in particular in wtFtL homopolymers (Fig. 3a, Supplementary Fig. S2b–e), severely impaired iron uptake of wtFtL homopolymers and significantly decreased iron incorporation in MtFtL/3FPM and FtL p.F167*/3FPM homopolymers (Fig. 3b,c, Supplementary Fig. S2f–i). Our data suggest that in HF, ferritin aggregation is a toxic gain-of-function related to the presence of the mutant sequence in MtFtL, while abnormal iron uptake/release is a loss-of-function related to the disruption of the structure of 4FPs. In iron release kinetic experiments, we observed an initial fast iron release phase lasting for 3 min in the wtFtL subunit. However, in MtFtL and FtL p.F167* homopolymers with active or inactive 3FPs, this initial fast iron-release phase was lengthened up to 8 min and the total amount of iron released was highly increased 4–7-fold (Fig. 4a). Interestingly, wtFtL homopolymers after the fast release phase showed a second, slower but sustained release phase (Fig. 4a,b (line plot)), a multiphasic kinetic also observed in other studies^29,32^. This second phase was also present, although at lower level, in wtFtL/3FPM mutant homopolymers, but was absent when the 4FPs were compromised. The lack of a second phase in these homopolymers may be due to the lack of iron, since most of the stored iron was already depleted during the first phase. In summary, normal 4FPs are necessary to retain iron and prevent iron from leaking from the mineralized core of ferritin. An open pore may allow the access of reducing agents and the fast removal of iron, enhancing the initial iron release phase. We also evaluated the effect of pH, ranging from 6.8 to 7.4, on the iron loading capacity (1000 Fe^2+^ atoms per ferritin molecule) of the different ferritin isoforms with non-functional 3FPs. We observed a significant decrease in iron storage capacity of homopolymers made by MtFtL and FtL p.F167* subunits with a mutated 3FP, even by small changes in the pH (from 7.4 to 7.2), without evidence of ferritin aggregation (Supplementary Fig. S3). These changes in iron storage were most likely due to a decreased capacity of the mutant nanocage to both iron uptake in the absence of functional 3FPs and iron retention of the mineralized core with abnormal 4FP, highlighting the importance of proper iron storage under acidic conditions.

### Ferritin precipitation and compromised iron storage capacity is a function of the number of MtFtL subunits

**Figure 6:**
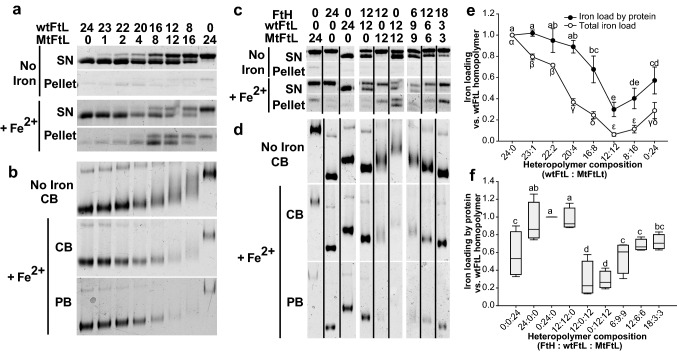
Heteropolymer assemble and function. WtFtL and MtFtL were disassemble and reassemble in the following wt to Mt ratios: 24:0, 23:1, 22:2, 20:4, 16:8, 12:12, 8:16, 0:24. 1 μM of heteropolymer was iron loaded with 1 mM Fe^2+^, or buffer (no iron, control) in 0.1 M HEPES buffer (pH 7.4) for 2 h. (**a**) SDS-PAGE electrophoresis of 10 μg of protein previously separated into supernatant (SN) and pellet (P) at 10 K g, stained with Coomassie Blue (CB). (**b**) Native PAGE electrophoresis of heteropolymers (SN fraction) stained with CB or Prussian Blue (PB). Iron content was quantified (**e**) as total iron (open circles) and iron normalized by protein (PB/CB signal) (filled circles). FtH, wtFtL and MtFtL were disassemble and reassemble in the following proportions (FtH:wtFtL:MtFtL): 0:0:24, 24:0:0, 0:24:0, 12:12:0, 12:0:12, 0:12:12, 6:9:9, 12:6:6 and 18:3:3. Heteropolymers were then iron-loaded by mixing 1 μM of 24mer with 1 mM Fe^2+^, or buffer (no iron, control) in 0.1 M HEPES buffer (pH 7.4) for 2 h. (**c**) 10 μg of protein were separated into SN and P at 10 K g, resolved by SDS-PAGE electrophoresis and stained with CB. (**d**) Native PAGE electrophoresis of heteropolymers (SN fraction), stained with CB or PB. Iron content was quantified (**f**) as iron normalized by protein (PB/CB signal). Results are presented as means + /-SEM. Groups with no coincident letters are statistical different (p < 0.05). Cropped gels are shown in (**c**) to improve the clarity of the presentation. Full-length gels are presented in Supplementary Figure S6. The figure was generated using Statgraphics Centurion XV v.15.1.02 https://www.statgraphics.com/, SigmaPlot for Windows v.12.5 Build 12.5.0.38 http://www.sigmaplot.com and Adobe Illustrator CC2019 23.0.3 https://www.adobe.com/products/illustrator.html.

To explore the effect(s) of the disruption of the E-helix in the FtL subunit in the context of the ferritin molecule, we assembled heteropolymers with various ratios of the mutant isoform subunit. Assessing heteropolymers may better reflect the in vivo situation in cells of patients with HF, where the ferritin moiety comprises a distribution of molecules containing wild-type and mutant isoforms in different ratios^8^. Purified and de-metalized homopolymers (self-assembled during purification) were disassembled into monomers under acid and denaturing conditions. Then, the isoforms wt:MtFtL were mixed at different subunit ratios (24:0; 23:1; 22:2; 20:4; 16:8; 12:12; 8:16 and 0:24) under progressive renaturing conditions to allow the subunits to re-assemble as heteropolymers. We assessed whether heteropolymers had the appropriate molar ratios by denaturing SDS-PAGE gels (Fig. 6a, top panel) and whether ferritins were soluble and assembled properly by native-PAGE (Fig. 6b, top panel, Supplementary Fig. S4a). We observed that heteropolymeric ferritin was resolved as a broader band rather than as a sharp band upon increasing the number of MtFtL isoforms (Fig. 6b, top panel). We believe this was a reflection of a wider ratio distribution of wt:Mt molecules present in each mixture, peaking at the expected ratio. To assess iron loading, apo-heteropolymers were loaded with 1000 Fe^2+^ atoms per ferritin molecule (Fig. 6a). Interestingly, at a ratio of just 23:1 (wt:Mt), we started to observe ferritin precipitation, becoming highly significant at a 22:2 ratio (Fig. 6a lower panel, Supplementary Fig. S4a). At a ratio of 16:8 (wt:Mt), heteropolymers showed also a significant decrease in iron storage capacity (Fig. 6b,e). Iron-loaded ferritin molecules had different mobility in native gels compared to the mobility of apo-heteropolymers, closer to the mobility of wtFtL homopolymer (Fig. 4b, Supplementary Fig. S4b), probably due to an enrichment in the soluble fraction of heteropolymers with higher amount of the wt monomers, while polymers with higher content of MtFtL subunits were more prone to precipitate. Altogether, we conclude that ferritin precipitation and compromised iron storage capacity was a function of the number of MtFtL subunits in the heteropolymer, emphasizing the deleterious effect of the mutant subunit in the ferritin molecule. Upon increasing the number of MtFtL subunits, the capacity of ferritin to retain mineralized iron diminished (Supplementary Fig. S4c), increasing significantly the total iron released at a 16:8 (wt:Mt) ratio, without any major changes in molecular assembly with lower proportion of mutant monomers per molecule (Supplementary Fig. S4d). This enhanced iron release was due largely to an augmented second phase of the release reaction, where iron seems to continuously leak from the ferritin core. The observed differences in the iron release kinetics between the disassembled and re-assembled mutant and wtFtL homopolymers and the corresponding self-assembled homopolymers (Fig. 4) may be due to alterations in the quaternary structure of the nanocage caused by the process of disassembly and reassembly of the monomers to form the polymers. The acidic disassembly of ferritin and later reassembly bringing the protein to neutral pH has been reported to not fully restore the structure of the nanocage^33^. This could account for the differences observed in the kinetic behavior of the reassembled polymers, highlighting the importance of studying the behavior of ferritin polymers assembled in the exact same conditions.

### Heteropolymers containing ferritin heavy chains do not show significant improvement of ferritin function

Cellular ferritin contains different ratios of FtL and FtH chains depending on the specific cell type, with variations in ferritin subunit composition affecting the rates of iron uptake and release^34^. We assessed whether the presence of FtH subunits in the ferritin nanocage could modify the stability and functionality of ferritin containing MtFtL subunits. We generated heteropolymers composed by FtH, wtFtL and MtFtL. Homopolymers (FtH:wtFtL:MtFtL at 0:0:24; 24:0:0; 0:24:0 ratios) were assembled as controls. Heteropolymers containing two of the subunits (FtH:wtFtL:MtFtL at 12:12:0; 12:0:12; 0:12:12 ratios) and with the three subunits with increasing amounts of FtH but equivalent amounts of wtFtL and MtFtL (FtH:wtFtL:MtFtL at 6:9:9; 12:6:6; 18:3:3 ratios) were assembled. The molar ratios of the apoferritins was assessed by SDS-PAGE (Fig. 6c) and their correct assembly and solubility by native-PAGE (Fig. 6d). Upon iron loading with 1000 Fe^2+^ atoms per ferritin molecule, we observed that the presence of the FtH subunit did not protect ferritin heteropolymers from the deleterious effects of the MtFtL subunit. Ferritin solubility and iron loading capacity, either total or by ferritin molecule, was similar in heteropolymers containing the MtFtL subunit with either wtFtL or FtH subunits at a 12:12 ratio (Fig. 6c,d,f; Supplementary Fig. S5a,b). Interestingly, assessment of iron release from the ferritin core using reducing agents indicated that a combination of MtFtL and FtH subunits had a diminished capacity in retaining iron in the core (Supplementary Fig. S5c,d) compared to heteropolymers composed by the wtFtL and MtFtL subunits (Supplementary Figs. S4, S5). This result is in agreement with the main role of FtL subunits, which do not have catalytic activity but are required for maintaining the stability of the ferritin complex that regulates iron storage and release^8,15,34^. Moreover, heteropolymers containing three subunits with increasing amounts of FtH subunits but equivalent amounts of wtFtL and MtFtL subunits (0:12:12; 6:9:9; 12:6:6; and 18:3:3 ratios) showed no significant improvement of ferritin function in terms of iron loading capacity, solubility or iron retained in the ferritin core (Fig. 6; Supplementary Figs. S4, S5). Although the mutant isoform has been reported to have an impact in the thermal stability of heteropolymers without altering the nanocage functionality^35^, we observed that the presence of just a few subunits of MtFtL in combination with wtFtL and/or FtH has a profound effect in ferritin function. The observed ferritin loss-of-function, even in polymeric ferritin, provide a rationale for a positive feed-back loop in HF, in which the failure of ferritin in its iron store function may lead to an increase in the levels of intracellular iron, increased translation of ferritin mRNAs, ferritin accumulation and iron overload^36^.

In conclusion, our work provides sound structural proof that mutagenesis of Asp-128 and Glu-131 in the 3FP of FtL subunits alters ferritin function without altering cage structure or other functions such as catalytic ferroxidation. Interfering with 3FPs function in the presence of altered 4FPs led to a diminished ferritin function in terms of iron loading capacity, solubility, and iron retained in the ferritin core. In vivo, a decrease in the 3FP functionality could lead to further increased in LIP and formation of IBs in patients with HF. We were able to assess for the first time the orientation of the C-terminus of the MtFtL subunit (last C-terminal 26 amino acids), which adopts opposite orientation compared to wtFtL. This sequence of MtFtL, capable to interact with other C-termini and facilitate iron-mediated aggregation of ferritin and formation of IBs in HF, remained previously unaccounted for by crystallography^9^. We also determined that the presence of MtFtL subunits at a very low ratio significantly compromises heteropolymeric ferritin solubility, providing rationale for the presence of IBs in numerous cell types in the CNS and in cells of systemic organs in patients with HF^5^. Finally, this work goes beyond the study of a particular neurodegenerative disease since ferritins are also widely exploited in bio-nanotechnology applications. Our ability to use ferritin to deliver concentrated doses of a particular compound^37^, avoiding drug loss across ferritin pores, may rely on a better understanding of the role of amino acid modifications and the structure of 3FPs and 4FPs of ferritin.

## Materials and methods

### Cloning and expression of ferritin polypeptides

Cloning, expression and purification of recombinant human ferritin isoforms and variants was carried out as previously described^7^. We expressed ferritin heavy chain (FtH), ferritin light chain (wtFtL), FtL with the D187I and E131F double mutation in the 3FP (3FPM), the FtL p.Phe167SerfsX26 variant (MtFtL), MtFtL with the 3FPM, the FtL p.F167* variant, and FtL p.F167* with the 3FPM. Purified homopolymers were run in a native PAGE (6%) to assess ferritin assemble and presence of possible aggregates above the expected size (Supplementary Fig. S1e).

### Transmission electron microscopy (TEM)

TEM was carried out as described^7^ to assess proper assemble of all the isoforms and measure the diameter of the nanocage. Briefly, ferritins were fixed using the “single droplet” parafilm protocol. The specimens were dropped onto a 400-mesh carbon/Formvar-coated grid (Nanoprobes) and allowed to absorb to the Formvar for a minimum of 1 min. Excess fluid was removed using filter paper, the unbound protein was washed and the grids were placed on a 50 µl drop of Nanovan (Nanoprobes) with the section side downwards. Finally, the grids were dried, placed in the grid chamber, and stored in desiccators before the samples were observed with a Tecnai G2 12 Bio Twin (FEI) transmission electron microscope.

### Preparation of apoferritins

To de-metallize purified ferritin proteins, all buffers were treated with chelex 100 resin (50–100mesh) for 48 h. Purified recombinant homopolymers were added to a dialysis bag (Spectra-Por 4, 12–14 K; Cole-Parmer) and dialyzed against 0.1 mM Acetate (pH4.5), 1 mM EDTA, 1% thioglycolic acid and 0.03% 2,2′-bipyridine for 18 h at 4 °C, followed by dialysis against 0.1 M HEPES buffer (pH 7.4) twice for 10–16 h each. We ensured the lack of iron by measuring that the decay of 265 nm absorbance was less of 0.5% over 15 min in the presence of 0.1 μM ascorbate. After assessing protein concentration of the de-metalized ferritins (Bradford method, BioRad), protein samples were aliquoted and stored at − 80 °C until further use.

### Cryo-EM sample preparation and data acquisition

Homopolymers of wtFtL and FtL p.F167* variant (50 µg/ml) were applied to a graphene oxide coated UltraAufoil R0.6/1 300 mesh grid (Quantifoil Micro Tools, Germany). Homopolymers of wtFtL/3FPM (50 µg/ml) and MtFtL/3FPM (100 µg/ml) were applied to a 0.01% chitosan coated lacey carbon grid (Ted Pella, USA) with graphene oxide. The sample grids were frozen using a Cryoplunge 3 system (Gatan, USA), then imaged on a 300 kV Titan Krios electron microscope (Thermo Fisher Scientific, USA) equipped with a post-GIF K2 summit camera (Thermo Fisher Scientific, USA). The movies were automatically collected with the software Leginon^38^. The imaging conditions of the four datasets are listed in Supplementary Table S1. Some of the movies of the wtFtL/3FPM dataset were collected with an additional 20 eV slit Quantum energy filter (Thermo Fisher Scientific, USA).

### Image processing

The raw movies were aligned and dose-weighted with Motioncor2/1.0.5^39^. The CTF stimations were conducted with Gctf-v1.06^40^ within RELION^41^. For the FtL p.F167* variant dataset, 214,168 particles were picked from 440 movies and were subjected to 2D and 3D classifications in RELION/2.1. Final 132,222 particles were selected and refined to 2.86 Å resolution and further refined to 2.5 Å resolution with JSPR^42^. The other three datasets were processed with the same procedures as those of the FtL p.F167* variant with details listed in Supplementary Table S1.

### Iron loading

For iron loading experiments, stock proteins were thawed on ice and centrifuged at 16,000*g* to eliminate any aggregates. Soluble proteins were incubated at a concentration of 1 μM of 24mer (unless otherwise indicated) with ferrous ammonium sulfate (within 0–4 mM) in 0.1 M HEPES buffer (pH 7.4, or at indicated pH) at room temperature for 2 h. Then the mixture was centrifuged at 16,000*g* to separate a soluble iron loaded protein fraction (SN) from the insoluble fraction (P). Samples were resolved by 16% acrylamide Tricine SDS-PAGE electrophoresis using denaturing conditions and in native conditions using 5% acrylamide native PAGE gels. Gels were stained with Coomassie blue (SimplyBlue SafeStain, Invitrogen) to detect total protein or Prussian blue [1% HCl, 1% K_4_(CN_6_)Fe freshly prepared, overnight in dark] to detect iron content in the protein.

### Preparation of heteropolymers

Apoferritin homopolymers were diluted to 0.5 mg of protein per ml in dialysis bags with 0.1 M Phosphate buffer (pH 7.4) and dialyzed against denaturation buffer [6 M Guanidine Hydrochloride, 0.1 M Phosphate (pH 2.5)] twice (16 h and 6 h changing buffer) at room temperature. Samples were collected and protein concentration determined. Samples were stored at − 80 °C until further use. To prepare heteropolymers, each isoform was diluted to a concentration of 24 μM with denaturing buffer and mixed at the desired proportions of each isoform. The 24mer were reassembled by diluting the mixture 10-fold with 0.1 M Phosphate buffer (pH 7.4) and 1 mM 1,4-Dithiothreitol, rotating for 6 h at room temperature and then dialyzed against 0.1 M HEPES buffer (pH 7.4) for 5 times during 48 h at 4 °C. Samples were concentrated (Amicon Ultra-4 filters) to approximately 2 μM of the 24mer. Protein concentration was assessed, and samples were stored at − 80 °C until further use.

### Iron removal from ferritin homopolymers

Iron release assays from iron loaded ferritins were performed as previously described^7,32^. Briefly, ferritins (2 μM) were loaded with 500:1 iron:ferritin overnight and the 16,000*g* supernatant was diluted 1:1 with iron removal buffer (0.1 M HEPES (pH 7.4), 5 mM NADH, 5 mM 2,2′-bipyridil, 5 mM Riboflavin 5′monophosphate). Iron release was determined as the increment in absorbance at 522 nm for 30 min.

## Supplementary information

Supplementary Information S1.

Supplementary Information S2.

## Data Availability

Cryo-EM maps have been deposited in the Electron Microscopy Data Bank (EMDB) under accession numbers EMDB-20225, EMDB-20227, EMDB-20228, EMDB-20229. Cryo-EM images have been deposited in the Electron Microscopy Public Image Archive (EMPIAR) under accession number EMPIAR-10263.
