# Supplementary material for: Cryo-EM structures and functional characterization of homo- and heteropolymers of human ferritin variants
Source: Sci Rep. 2020 Nov 26;10:20666. doi: 10.1038/s41598-020-77717-4 (PMC7692541; doi:10.1038/s41598-020-77717-4)
Supplement: Supplementary file 1 — Supplementary Information S1. [file 41598_2020_77717_MOESM1_ESM.docx]

**Sup. Figures**

**Sup Fig. 1.** Ribbon representations of ferritin homopolymeric (24-subunit) structures viewed down one of the 3-fold axes (a) and the 4-fold axes (b). The ferritin subunits are colored differently for emphasis. Single subunit (c) highlighting the short (red) E-helix, which forms the sides of 4FPs. (d) Sequence alignment of wtFTL, MtFTL and FtL p.F167* polypeptides starting at aa 121. The helical domains D and E are indicated above their respective sequences (Protein Data Bank code 2fg4). The MtFTL sequence has a C terminus that is altered in sequence and length (9). FtL p.F167* polypeptides have a premature stop codon (p.F167*) that disrupts the E helical domain. The D128 and E131 aa are indicated in red. Purified recombinant apoferritin homopolymers were separated by native PAGE (e). Homopolymers were assemble using recombinants with a normal (N) or with the D127I and E130F 3FP aa mutations (M). Gel was stained by Coomassie blue and it is shown in greyscale color. Molecular weight markers are: bovine thyroglobulin (669KDa), horse ferritin (443Kda) and sweet potato beta-amylase (200KDa). Ultrastructural characterization of ferritin homopolymers by TEM of wtFtL with 3FP mutations (f), MtFtL with 3FP mutations (g), and FtL p.F167* with 3FP mutations (h). The dark cores most likely represent Nanovan that has penetrated the interior of the 24-mers. Scale bar, 50 nm. Pictures (a-c) were made from crystallographic structures in the RCSB database using files 2fg8 and 2ffx. a and b were produced using SPDB-Viewer and rendered with Pov-Ray. c was produced using PyMol for Windows. The figure was generated using Adobe Illustrator CC2019 23.0.3 https://www.adobe.com/products/illustrator.html

**Sup Fig. 2.** Soluble homopolymers were quantified from 3 independent experiments comparing homopolymers of wtFTL (circles), MtFtL (diamonds) and FtL p.F167* (triangles) with intact 3FP (filled symbols) (a) and homopolymers with the 3FP mutations (b). Significant differences were observed between wtFtL compared to wtFtL/3FPM (c), with the wtFtL compared to MtFtL and to MtFtl/3FPM (d), and with the FtL p.F167* with the 3FP mutated (e). The ratio of iron content to ferritin protein from 3 independent experiments was calculated (PB/CB signal) upon increasing iron load (f) and at 1.5mM FAS (g). Total iron content (PB signal) was calculated upon increasing iron load (h) and at 1.5 mM FAS (i). Results are presented as mean ± SEM. Symbols represent statistical significance comparing 2 groups as described in each graph (p<0.05). G & I: groups with no coincident letters that are statistical different (p<0.05). The figure was generated using Statgraphics Centurion XV v.15.1.02 https://www.statgraphics.com/, SigmaPlot for Windows v.12.5 Build 12.5.0.38 http://www.sigmaplot.com and Adobe Illustrator CC2019 23.0.3 https://www.adobe.com/products/illustrator.html

**Sup Fig. 3.** Sensitivity of iron loading to pH. (a) Ferritin homopolymers were iron loaded (1:1000 ratio) in HEPES buffer at pH 7.4, 7.2, 7.0 and 6.8. Then the soluble ferritin was resolved in a native PAGE and stained for protein (coomassie blue, CB, top row) and iron content (Prussian blue, PB, bottom row). MM, Precision Plus Protein All Blue protein standard (Bio Rad). (b) The ratio iron to protein (PB to CB signal) was calculated from 3 independent experiments. WtFTL was represented with a circle, MtFTL with a diamond and FtL p.F167* with a triangle. Homopolymers with a normal 3FP were represented with filled symbols while homopolymers with mutated 3FP were represented with open symbols. Results are presented as mean ± SEM. Groups with no coincident letters are statistical different (p<0.05). The figure was generated using Statgraphics Centurion XV v.15.1.02 https://www.statgraphics.com/, SigmaPlot for Windows v.12.5 Build 12.5.0.38 http://www.sigmaplot.com and Adobe Illustrator CC2019 23.0.3 https://www.adobe.com/products/illustrator.html

**Sup Fig. 4.** Heteropolymers of wt and MtFTL were assessed for iron loading and release. (a) Quantification of the fraction of iron loaded protein in the supernatant fraction (SDS-PAGE) indicated in Figure 6a. (b) Quantification of ferritin (native PAGE) for total iron loading (open circles) and iron loading normalized by protein (PB/CB signal) (filled circles). (c) The relative mobility of ferritin in CB stained native gels was calculated relative to a marker, and normalized towards the non-iron loaded protein (apoferritin) for each heteropolymer. A value >1 indicate that the holoferritin has increased electrophoretic mobility compared to the apoferritin. (d) After iron loading (500:1 Fe^2+^:ferritin), an aliquot of the heteropolymers was subjected to iron release challenge by reducing agents for 30 minutes, monitoring the Fe^2+^ release as a function of 520 nm absorbance. (e) The total iron released was plotted (Grey bars, left axis) as was the iron released during the second release phase from 10 to 30 minutes (black circles, right axis). All data come from 4 independent experiments and are presented as means ± SEM. Different symbols (letters) denote statistical significance (p<0.05). The figure was generated using Statgraphics Centurion XV v.15.1.02 https://www.statgraphics.com/, SigmaPlot for Windows v.12.5 Build 12.5.0.38 http://www.sigmaplot.com and Adobe Illustrator CC2019 23.0.3 https://www.adobe.com/products/illustrator.html

**Sup Fig. 5.** Iron loading and release of ferritin heteropolymers. (a) FtH, wtFtL and MtFtL heteropolymers were iron-loaded, separated into supernatant and pellet at 10kg, resolved by SDS-PAGE, stained with CB as indicated in Fig.6c and the fraction of iron loaded protein in the supernatant was quantified. (b) Quantification of heteropolymer’s total iron loading as indicated in Fig.6d. (c) After iron loading 500:1 Fe2+:ferrritin, an aliquot of the heteropolymers was subjected to iron release challenge by reducing agents for 30 minutes. Fe^2+^ release was monitored as a function of 520nm absorbance. (d) Total iron released (Grey bars, left axis) and iron released by time and protein during the second phase (from 10 to 30 minutes) (black circles, right axis). All data come from 4 independent experiments and are presented as means ± SEM. Different symbols (letters) denote statistical significance (p<0.05). The figure was generated using Statgraphics Centurion XV v.15.1.02 https://www.statgraphics.com/, SigmaPlot for Windows v.12.5 Build 12.5.0.38 http://www.sigmaplot.com and Adobe Illustrator CC2019 23.0.3 <https://www.adobe.com/products/illustrator.html>

**Sup Fig. 6.** Full-length gels for Fig 3. MM, Precision Plus Protein All Blue protein standard (Bio Rad). The figure was generated using Adobe Illustrator CC2019 23.0.3 <https://www.adobe.com/products/illustrator.html>

**Sup Fig. 7.** Full-length gels for Fig 6. Gel contrast was enhanced solely to reveal very faint bands. For quantifications, the unmodified original images were used. (a) Original gels of supernatant (SN) samples used for Fig6a (the area used is highlighted in a red box) and Fig6c (each lane used is highlighted in cyan boxes). (b) Original gels of pellet (P) samples used for Fig6a (the area used is highlighted in a red box) and Fig6c (each lane used is highlighted in cyan boxes). (c) Original native gels used for Fig6b where the area used is highlighted in a red box. (d) Original native gels used for Fig6d where each lane used is highlighted in red boxes. Gels were stained with Commassie blue (CB) or Prussian Blue (PB). Proteins were loaded with iron (+Fe2+) or without iron (No Iron). The relative composition of each heteropolimer is described on top of each panel. MM lane correspond to molecular marker. Cropped areas used in Fig6 are depicted inside rectangles in each gel.The figure was generated using Adobe Illustrator CC2019 23.0.3 https://www.adobe.com/products/illustrator.html

**Sup Fig. 8.** Full-length gels for Fig 6. Original unmodified contrast parameters used for quantification. (**a**) Original gels of supernatant (SN) samples used for Fig6a (the area used is highlighted in a red box) and Fig6c (each lane used is highlighted in cyan boxes). (**b**) Original gels of pellet (P) samples used for Fig6a (the area used is highlighted in a red box) and Fig6c (each lane used is highlighted in cyan boxes). (**c**) Original native gels used for Fig6b where the area used is highlighted in a red box. (**d**) Original native gels used for Fig6d where each lane used is highlighted in red boxes. Gels were stained with Commassie blue (CB) or Prussian Blue (PB). Proteins were loaded with iron (+Fe2+) or without iron (No Iron). The relative composition of each heteropolimer is described on top of each panel. MM lane correspond to molecular marker. Cropped areas used in Fig6 are depicted inside rectangles in each gel. The figure was generated using Adobe Illustrator CC2019 23.0.3 <https://www.adobe.com/products/illustrator.html>

**Supplementary Table 1.** **Cryo-EM data of wtFtL, FtLp.F167*, wtFtL/3FP, and** **MtFtL/3FP.**
