# Supplementary material for: Cryo-EM structures and functional characterization of homo- and heteropolymers of human ferritin variants
Source: Sci Rep. 2020 Nov 26;10:20666. doi: 10.1038/s41598-020-77717-4 (PMC7692541; doi:10.1038/s41598-020-77717-4)
Supplement: Supplementary file 2 — Supplementary Information S2. [file 41598_2020_77717_MOESM2_ESM.pdf]

Cryo-EM structures and functional characterization of homo- and heteropolymers of human ferritin variants

Jose Irimia-Dominguez[1*], Chen Sun[2], Kunpeng Li[2], Barry B. Muhoberac[3], Grace I. Hallinan[1], Holly J. Garringer[1], Bernardino Ghetti[1,4], Wen Jiang[2], Ruben Vidal[1,4,*]

[1]Department of Pathology and Laboratory Medicine, Indiana University School of Medicine, Indianapolis, IN 46202, USA
[2]Department of Biological Sciences, Markey Center for Structural Biology, Purdue University, West Lafayette, IN 47906, USA
[3]Department of Chemistry and Chemical Biology, Indiana University-Purdue University Indianapolis, Indianapolis, IN 46202, USA
[4]Stark Neurosciences Research Institute, Indiana University School of Medicine, Indianapolis, IN 46202, USA

SFig. 1

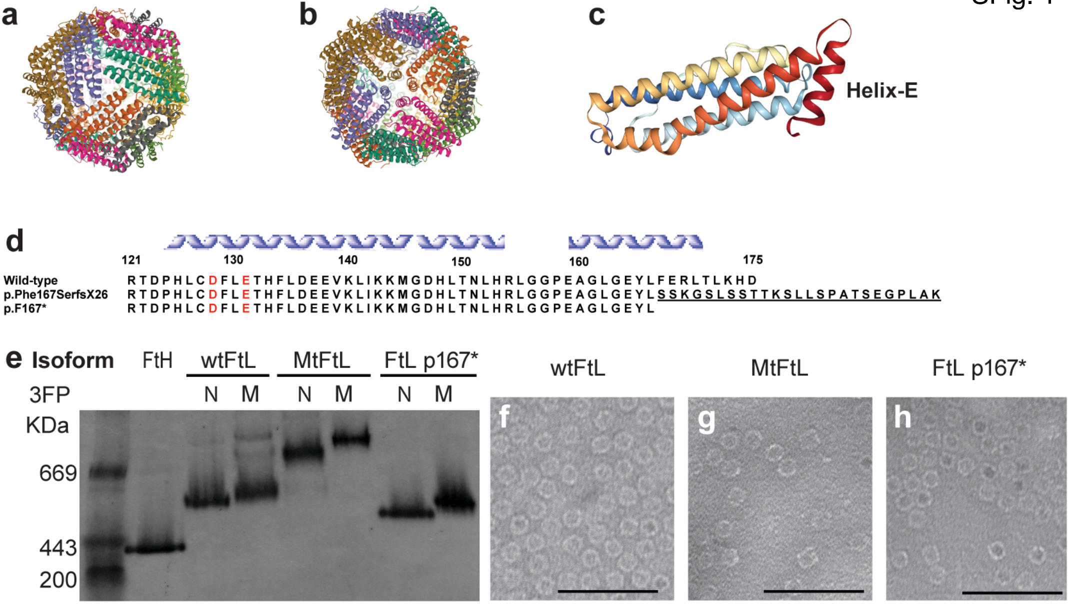
a
b
c
Helix-E
d
121
130
140
150
160
175
Wild-type RTDPHLCDFLETHFLDEEVKLIKKMGDHLTNLHRLGGPEAGLGEYLFERLTLKHD
p.Phe167SerfsX26 RTDPHLCDFLETHFLDEEVKLIKKMGDHLTNLHRLGGPEAGLGEYLSSKGSLSSTTKSLLSPATSEGPLAK
p.F167* RTDPHLCDFLETHFLDEEVKLIKKMGDHLTNLHRLGGPEAGLGEYL
e Isoform FtH wtFtL MtFtL FtL p167*
3FP N M N M N M
KDa
669
443
200
wtFtL
MtFtL
FtL p167*
f
g
h

SFig. 2

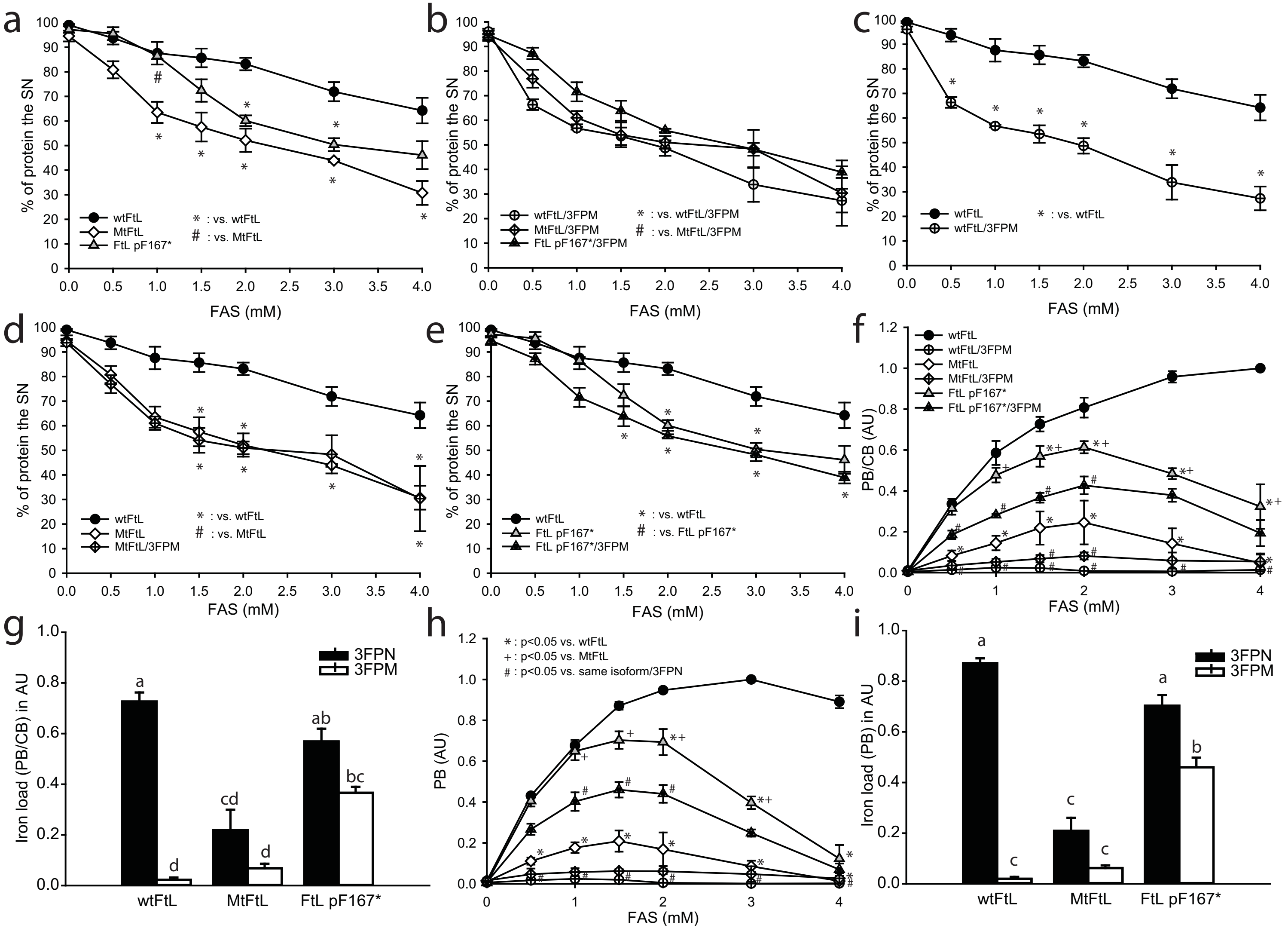
a
b
c
d
e
f
g
h
i
% of protein the SN
FAS (mM)
PB/CB (AU)
PB (AU)
Iron load (PB/CB) in AU
Iron load (PB) in AU
wtFtL
MtFtL
FtL pF167*
wtFtL/3FPM
MtFtL/3FPM
FtL pF167*/3FPM
* : vs. wtFtL
# : vs. MtFtL
* : vs. wtFtL/3FPM
# : vs. MtFtL/3FPM
# : vs. FtL pF167*
3FPN
3FPM
* : p<0.05 vs. wtFtL
+ : p<0.05 vs. MtFtL
# : p<0.05 vs. same isoform/3FPN

SFig. 3

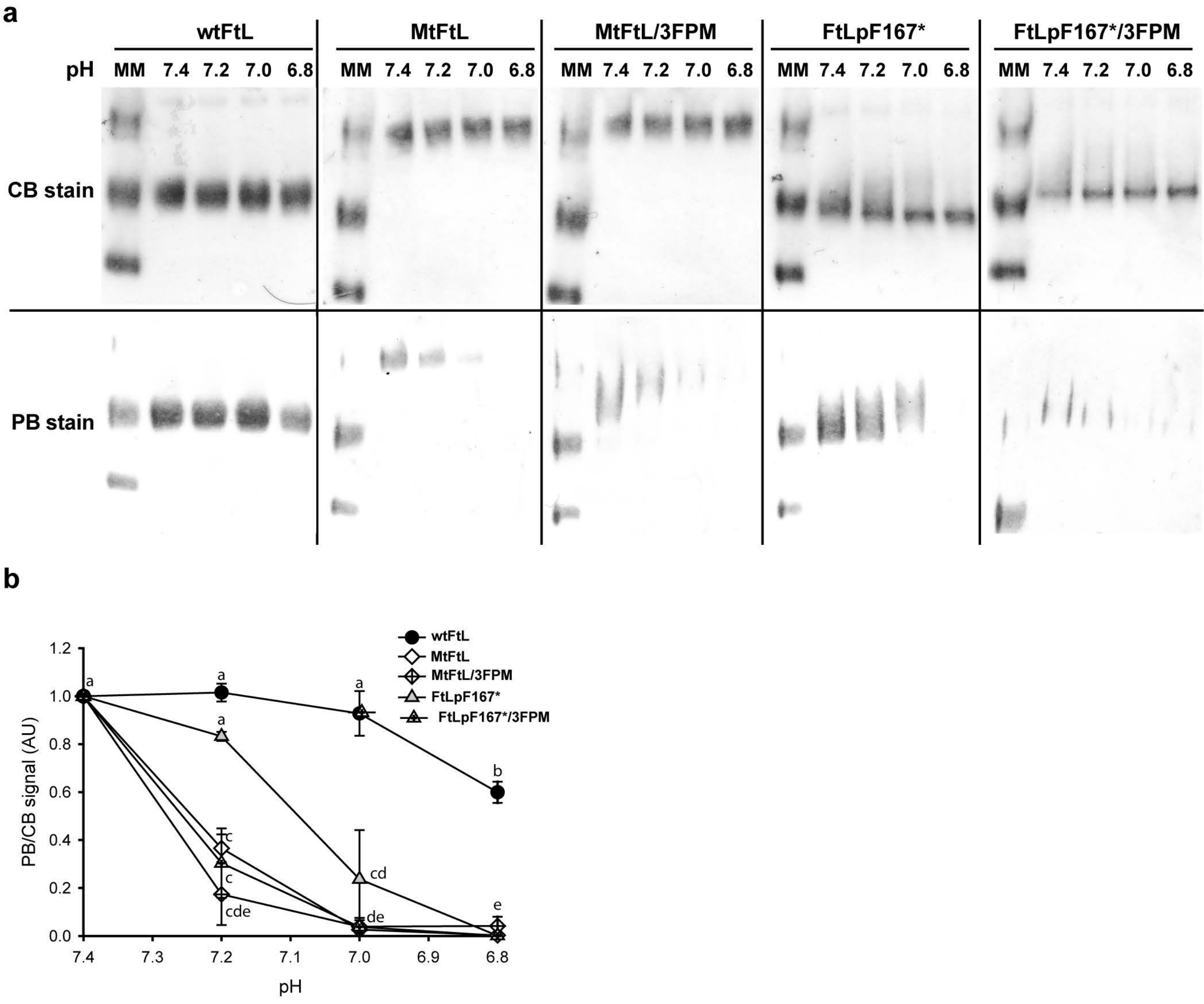
a
wtFtL
MtFtL
MtFtL/3FPM
FtLpF167*
FtLpF167*/3FPM
pH
MM 7.4 7.2 7.0 6.8
CB stain
PB stain
b
wtFtL
MtFtL
MtFtL/3FPM
FtLpF167*
FtLpF167*/3FPM
PB/CB signal (AU)
pH
a
b
c
cd
cde
de
e

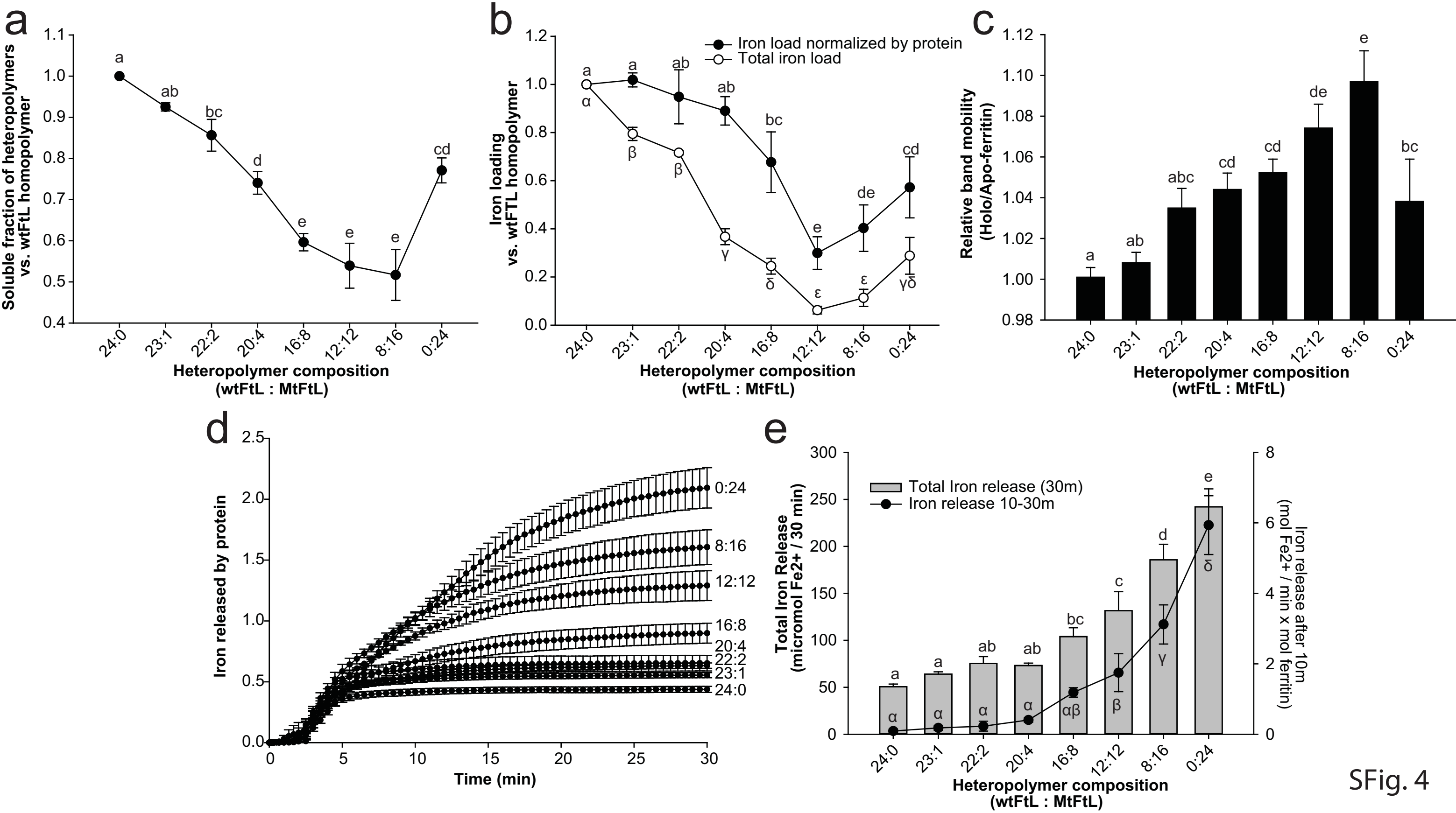
a
Soluble fraction of heteropolymers vs. wtFtL homopolymer
Heteropolymer composition (wtFtL : MtFtL)
b
Iron load normalized by protein
Total iron load
Iron loading vs. wtFTL homopolymer
Heteropolymer composition (wtFtL : MtFtL)
c
Relative band mobility (Holo/Apo-ferritin)
Heteropolymer composition (wtFtL : MtFtL)
d
Iron released by protein
Time (min)
0:24
8:16
12:12
16:8
20:4
22:2
23:1
24:0
e
Total Iron release (30m)
Iron release 10-30m
Total Iron Release (micromol Fe2+ / 30 min)
Iron release after 10m (mol Fe2+ / min x mol ferritin)
Heteropolymer composition (wtFtL : MtFtL)

SFig. 4

SFig. 5

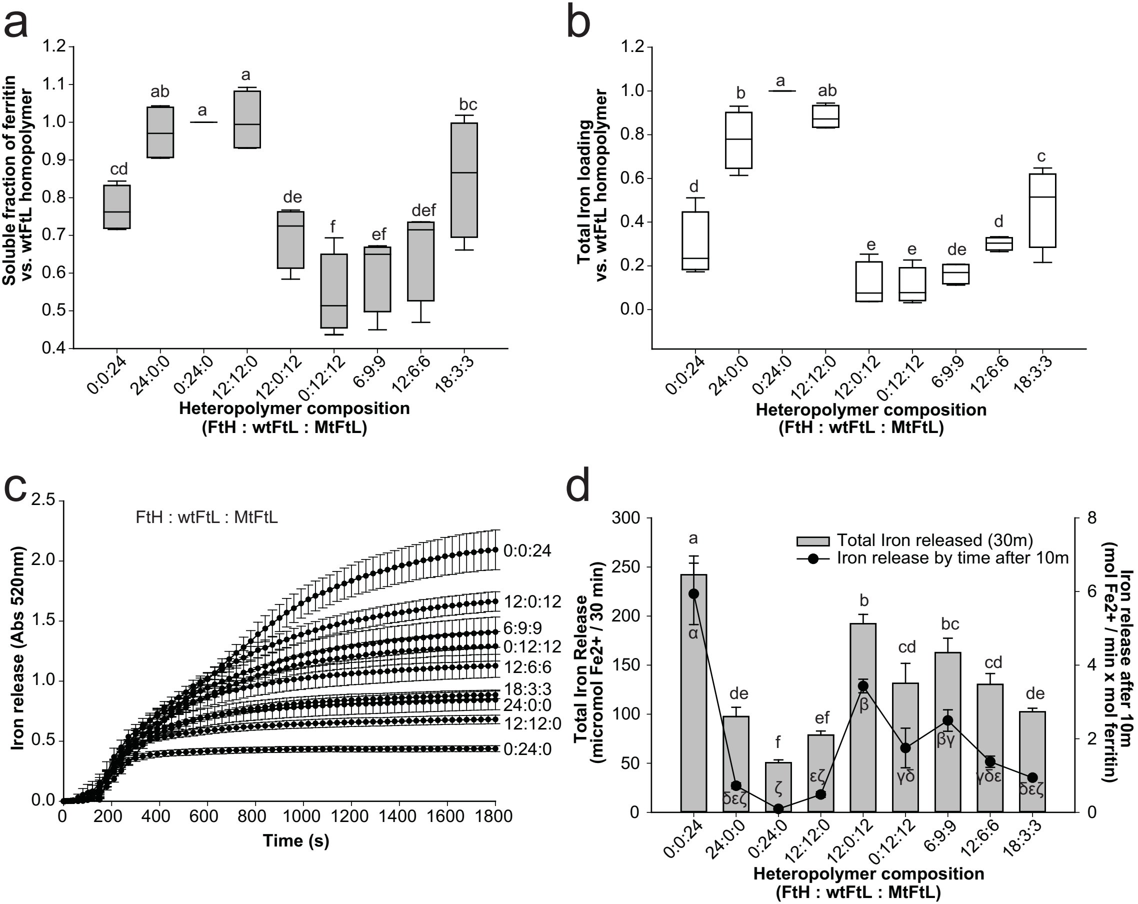

a
Soluble fraction of ferritin vs. wtFtL homopolymer
1.2
1.1
1.0
0.9
0.8
0.7
0.6
0.5
0.4
cd
ab
a
a
de
f
ef
def
bc
0:0:24
24:0:0
0:24:0
12:12:0
12:0:12
0:12:12
6:9:9
12:6:6
18:3:3
Heteropolymer composition
(FtH : wtFtL : MtFtL)
b
Total Iron loading vs. wtFtL homopolymer
1.2
1.0
0.8
0.6
0.4
0.2
0.0
d
b
a
ab
e
e
de
d
c
0:0:24
24:0:0
0:24:0
12:12:0
12:0:12
0:12:12
6:9:9
12:6:6
18:3:3
Heteropolymer composition
(FtH : wtFtL : MtFtL)
c
FtH : wtFtL : MtFtL
Iron release (Abs 520nm)
2.5
2.0
1.5
1.0
0.5
0.0
0:0:24
12:0:12
6:9:9
0:12:12
12:6:6
18:3:3
24:0:0
12:12:0
0:24:0
0
200
400
600
800
1000
1200
1400
1600
1800
Time (s)
d
Total Iron Release (micromol Fe2+ / 30 min)
300
250
200
150
100
50
0
Total Iron released (30m)
Iron release by time after 10m
Iron release after 10m (mol Fe2+ / min x mol ferritin)
8
6
4
2
0
a
de
f
ef
b
cd
bc
cd
de
α
δεζ
ζ
εζ
β
γδ
βγ
γδε
δεζ
0:0:24
24:0:0
0:24:0
12:12:0
12:0:12
0:12:12
6:9:9
12:6:6
18:3:3
Heteropolymer composition
(FtH : wtFtL : MtFtL)

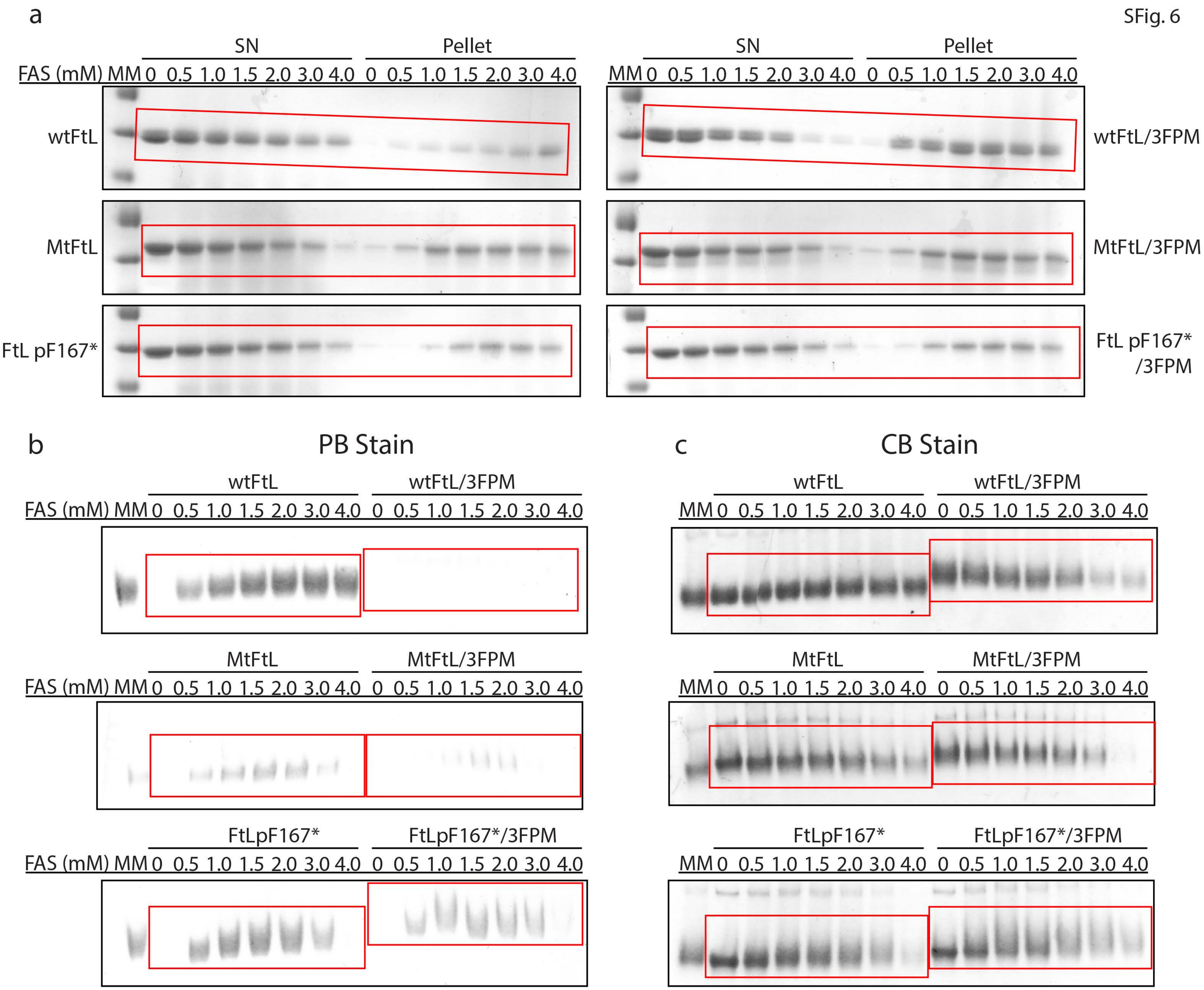
a
SN
Pellet
FAS (mM) MM 0 0.5 1.0 1.5 2.0 3.0 4.0 0 0.5 1.0 1.5 2.0 3.0 4.0
wtFtL
MtFtL
FtL pF167*
MM 0 0.5 1.0 1.5 2.0 3.0 4.0 0 0.5 1.0 1.5 2.0 3.0 4.0
wtFtL/3FPM
MtFtL/3FPM
FtL pF167* /3FPM
b
PB Stain
wtFtL
wtFtL/3FPM
MtFtL
MtFtL/3FPM
FtLpF167*
FtLpF167*/3FPM
FAS (mM) MM 0 0.5 1.0 1.5 2.0 3.0 4.0 0 0.5 1.0 1.5 2.0 3.0 4.0
c
CB Stain
wtFtL
wtFtL/3FPM
MtFtL
MtFtL/3FPM
FtLpF167*
FtLpF167*/3FPM
MM 0 0.5 1.0 1.5 2.0 3.0 4.0 0 0.5 1.0 1.5 2.0 3.0 4.0

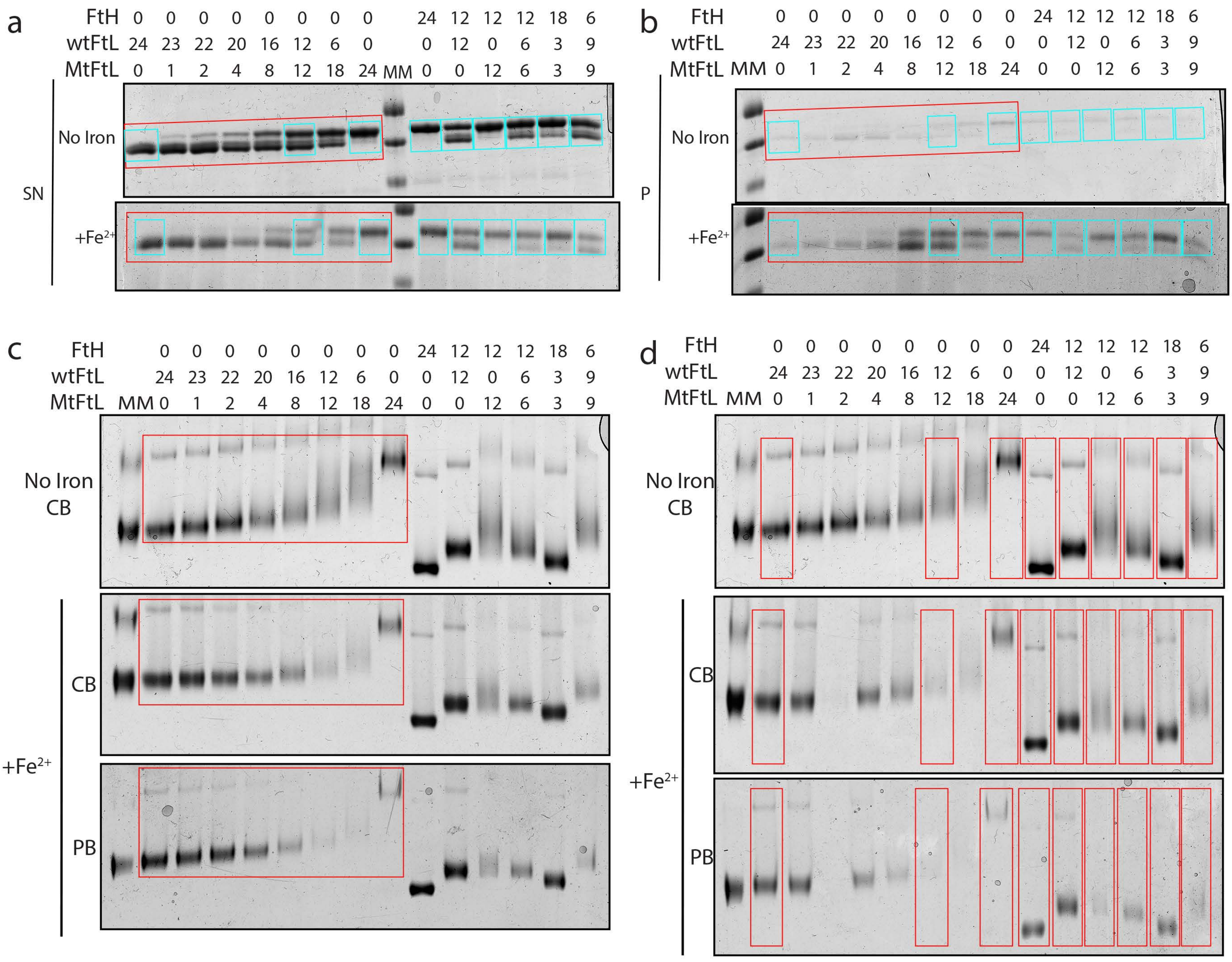
a
FtH 0 0 0 0 0 0 0 0 24 12 12 12 18 6
wtFtL 24 23 22 20 16 12 6 0 0 12 0 6 3 9
MtFtL 0 1 2 4 8 12 18 24 MM 0 0 12 6 3 9
No Iron
SN
+Fe2+
b
FtH 0 0 0 0 0 0 0 0 24 12 12 12 18 6
wtFtL 24 23 22 20 16 12 6 0 0 12 0 6 3 9
MtFtL MM 0 1 2 4 8 12 18 24 0 0 12 6 3 9
No Iron
P
+Fe2+
c
FtH 0 0 0 0 0 0 0 0 24 12 12 12 18 6
wtFtL 24 23 22 20 16 12 6 0 0 12 0 6 3 9
MtFtL MM 0 1 2 4 8 12 18 24 0 0 12 6 3 9
No Iron
CB
CB
+Fe2+
PB
d
FtH 0 0 0 0 0 0 0 0 24 12 12 12 18 6
wtFtL 24 23 22 20 16 12 6 0 0 12 0 6 3 9
MtFtL MM 0 1 2 4 8 12 18 24 0 0 12 6 3 9
No Iron
CB
CB
+Fe2+
PB

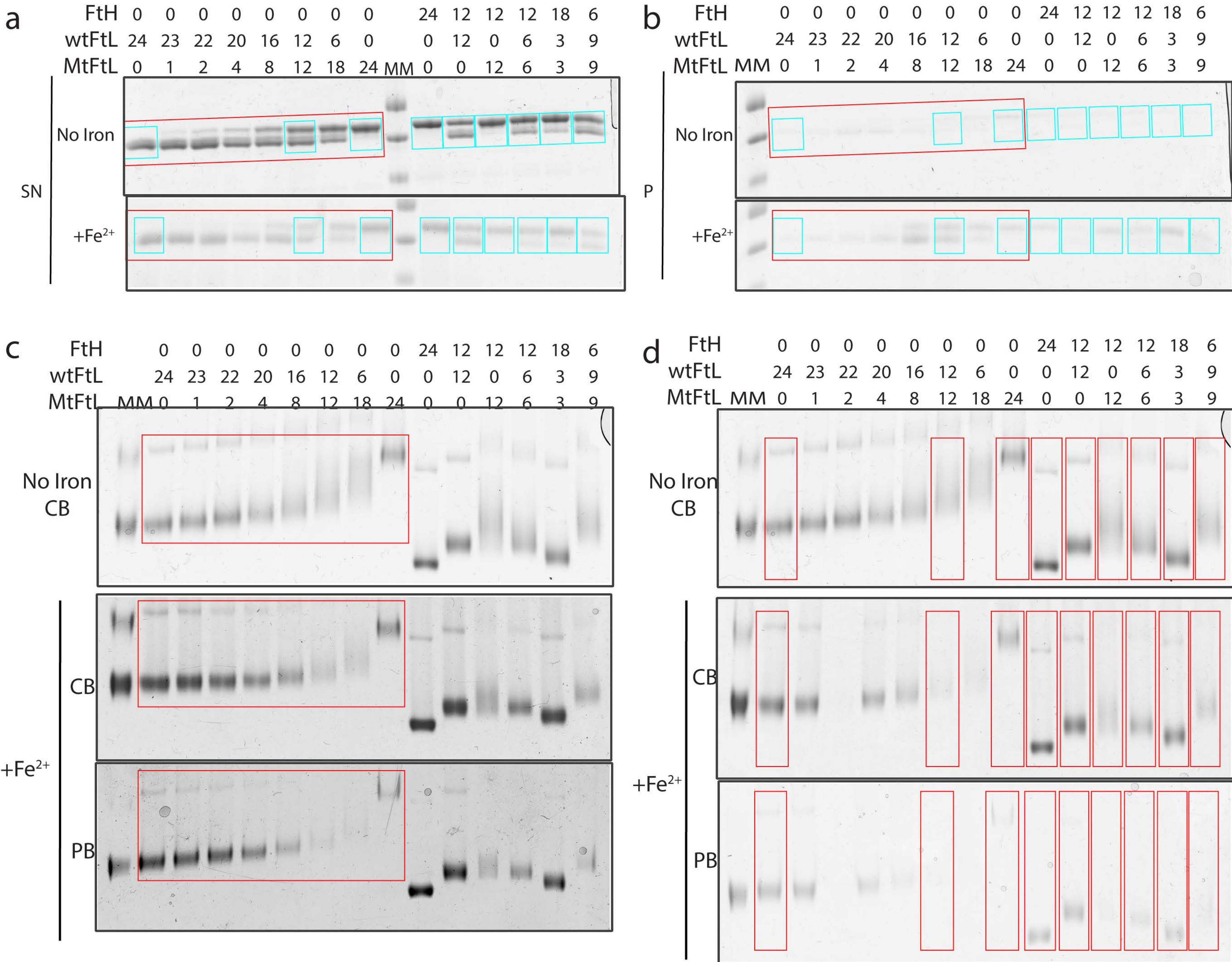
a
FtH 0 0 0 0 0 0 0 0 24 12 12 12 18 6
wtFtL 24 23 22 20 16 12 6 0 0 12 0 6 3 9
MtFtL 0 1 2 4 8 12 18 24 MM 0 0 12 6 3 9
No Iron
SN
+Fe2+
b
FtH 0 0 0 0 0 0 0 0 24 12 12 12 18 6
wtFtL 24 23 22 20 16 12 6 0 0 12 0 6 3 9
MtFtL MM 0 1 2 4 8 12 18 24 0 0 12 6 3 9
No Iron
P
+Fe2+
c
FtH 0 0 0 0 0 0 0 0 24 12 12 12 18 6
wtFtL 24 23 22 20 16 12 6 0 0 12 0 6 3 9
MtFtL MM 0 1 2 4 8 12 18 24 0 0 12 6 3 9
No Iron CB
CB
PB
+Fe2+
d
FtH 0 0 0 0 0 0 0 0 24 12 12 12 18 6
wtFtL 24 23 22 20 16 12 6 0 0 12 0 6 3 9
MtFtL MM 0 1 2 4 8 12 18 24 0 0 12 6 3 9
No Iron CB
CB
PB
+Fe2+

Supplementary Table 1

	wtFtL	FtLp.F167*	wtFtL/3FP	MtFtL/3FP
Sample concentration (µg/ml)	50	50	50	100
Grid type	UltraAufoil	UltraAufoil	Lacey carbon	Lacey carbon
Voltage (kV)	300	300	300	300
Total Dose (e/Å^2)	17.5	35	35	35
Nominal magnification	22,500	29,000	130,000	130,000
Energy filter	-	-	mixed	-
Volta phase plate	-	+	+	+
Pixel size (Å)	0.655	0.511	0.545	0.545
# of Movies	320	440	928	921
# of Frames	40	50	64	64
Particles picked	125,654	214,168	136,378	373,743
Final Particles	22,394	132,222	55,936	284,744
Resolution (Å)	3.5	2.52	2.56	2.54
